# The Epidemiology, Molecular, and Clinical of Human Adenoviruses in Children Hospitalized With Acute Respiratory Infections

**DOI:** 10.3389/fmicb.2021.629971

**Published:** 2021-02-16

**Authors:** Shunhang Wen, Zupan Lin, Yue Zhang, Fangfang Lv, Haiyan Li, Xueya Zhang, Li Lin, Hui-Hui Zhu, Zhi Xu, Changchong Li, Hailin Zhang

**Affiliations:** ^1^Department of Children’s Respiration Disease, The Second Affiliated Hospital and Yuying Children’s Hospital, Wenzhou Medical University, Wenzhou, China; ^2^Department of Pediatrics, The Third Affiliated Hospital, Wenzhou Medical University, Wenzhou, China; ^3^Ningbo Health Gene Technologies Ltd., Ningbo, China

**Keywords:** human adenovirus, molecular types, epidemiology, acute respiratory infections, children

## Abstract

**Introduction:**

Human adenovirus (HAdV) is a common pathogen in children with acute respiratory infections (ARIs). The aim was to describe the epidemiology, molecular, and clinical characteristics of HAdV among children hospitalized with ARIs in Wenzhou in southeastern China.

**Methodology:**

From January 2018 to December 2019, nasopharyngeal swab or sputum specimens were prospectively collected from hospitalized children with ARIs. HAdV was detected using direct immunofluorescence. We used a multiplex PCR assay combined with capillary electrophoresis targeting the hexon gene’s hypervariable region to identify HAdV types 1, 2, 3, 4, 5, 7, 14, 21, 37, 40, 41, and 55. We analyzed the epidemiological, molecular, and clinical data according to the HAdV type.

**Results:**

HAdVs were detected in 1,059 (3.5%) of the total of 30,543 children tested. A total of 947 cases with monotype HAdV identified by the PCR assay were included in the analysis. HAdV-3 (415/947, 43.8%), HAdV-7 (318/947, 33.6%), HAdV-2 (108/947, 11.4%), and HAdV-1 (70/947, 7.4%) were the predominant types. Of the 550 (58.1%) cases detected from December 2018 to August 2019, HAdV-3, and HAdV-7 were the main types. The main diagnoses included 358 cases of pneumonia, 232 cases of tonsillitis, 198 cases of bronchitis, and 159 cases of upper respiratory tract infection (URTI). Among children with pneumonia the main types were HAdV-7 (51.1%), HAdV-3 (36.9%), and HAdV-1 (2.2%). Among children with bronchitis, the main types were HAdV-3 (48.0%), HAdV-7 (28.3%), and HAdV-2 (10.6%). Among children with URTIs, the main types were HAdV-3 (49.7%), HAdV-7 (22.6%), and HAdV-2 (13.2%). Among children with tonsillitis, the main types were HAdV-3 (47.4%), HAdV-2 (22.4%), and HAdV-7 (18.5%). In total, 101 (55.2%) patients required supplemental oxygen, 15 (8.2%) required critical care, and 1 child (0.5%) with HAdV-7 pneumonia died.

**Conclusion:**

HAdV-3 -7, -2, and -1 were the predominant types identified in hospitalized children with ARIs in Wenzhou. From December 2018 to August 2019, there were outbreaks of HAdV-3 and -7. There were significant differences in HAdV types among children with pneumonia, tonsillitis, bronchitis, and URTI. HAdV-7 can cause more severe pneumonia in children than HAdV-3.

## Introduction

Human adenovirus (HAdV) is a major cause of acute respiratory infections (ARIs) in children. HAdV belongs to the *Mastadenovirus* genus, and is a non-enveloped double-stranded DNA virus. HAdV divided into seven different species (A to G), which consists of 103 HAdV types (HAdV-1 to HAdV-51 were serotypes and HAdV-52 to HAdV-103 were genotypes). HAdV species B (types 3, and 7) and species C (types 1, and 2) are most commonly associated with respiratory infections (Xu et al., 2018; Zhao et al., 2020).

Adenoviral respiratory infections can manifest as upper respiratory tract infection (URTI), tonsillitis, bronchitis, and pneumonia. As most adenoviral respiratory infections are of mild to moderate severity, HADV-3 and -7 can cause life-threatening infections and outbreaks (Yu et al., 2016; Xie et al., 2018a). Adenoviral infection is the leading cause of post-infectious bronchiolitis obliterans in children (Zhong et al., 2020). The prognosis of bronchiolitis obliterans is overall poor as a result of irreversible pulmonary fibrosis and airway obstruction. There is limited information available on the association of HAdV types and outcomes in children with severe adenovirus pneumonia.

Typing is currently not performed in most HAdV respiratory infections, and molecular investigations are generally only performed in large outbreaks or cases of unusual severity. However, typing of HAdV is important for understanding the local epidemiology and for vaccine development. Although the molecular epidemiology of adenoviral infections have reported in Guangzhou (Chen et al., 2016), Beijing (Xu et al., 2018), and Hunan (Xie et al., 2018a), China. However, the molecular epidemiology about HAdV among hospitalized children with ARIs is insufficient in the southeastern region of China. We conducted a prospective study to obtain data on the epidemiological, molecular, clinical characteristics and outcomes of HAdV infections among hospitalized children with ARIs in Wenzhou, southeastern China, from January 2018 to December 2019.

## Materials and Methods

### Study Design and Clinical Specimens

From January 2018 to December 2019, nasopharyngeal secretion specimens (NPSs) or sputum were collected from hospitalized children aged <18 years who presented with ARIs to The Second Affiliated Hospital and Yuying Children’s Hospital, Wenzhou Medical University, Zhejiang Province, China. Clinical syndromes of URTI, tonsillitis, bronchitis, and pneumonia were categorized as ARIs. Cases with HAdV detected by direct immunofluorescence were included. Approximately 1 mL of respiratory tract secretion was collected from each participant and stored at −80°C. Children with HAdV pneumonia were followed-up for 6 months by telephone and outpatient visits. Children whose guardians refused to provide demographic data, and those with insufficient specimen to perform adenovirus typing were excluded from the study. Data were collected on the clinical characteristics and outcomes from hospital database. The study was approved by the Ethics Committee of the Second Affiliated Hospital and Yuying Children’s Hospital, Wenzhou Medical University.

### Nucleic Acid Extraction

A total of 300 μL of DNA was extracted from each respiratory tract secretions sample using the Viral Total Nucleic Acid Extraction (Ningbo Health Gene Technologies Ltd., Ningbo, China) according to the manufacturer’s instructions, as described previously (Wen et al., 2019). The extracts were eluted into 80 μL of DNase-free water and stored at −80°C.

### Adenovirus Typing Assay

The adenovirus typing test kit (Ningbo Health Gene Technologies Ltd., Ningbo, China) was used to simultaneously detect HAdV types 1, 2, 3, 4, 5, 7, 14, 21, 37, 40, 41, and 55. Primer pairs targeting the 12 tested respiratory tract pathogens and three internal controls (human RNA control, human DNA control, and RT-PCR control) were amplified in a single tube, as described previously (Wen et al., 2019). Targeted hypervariable regions 1–6 of the hexon gene, HAdV were molecularly typed by nested PCR amplification and sequencing.

### The Diagnosis of Bronchiolitis Obliterans After Human Adenovirus Pneumonia

To diagnose bronchiolitis obliterans, a combination of medical history, clinical characteristics, pulmonary function test, and high resolution computerized tomography were used (Jerkic et al., 2020). The clinical signs are persistent wheezing or cough, tachypnea, dyspnea, exercise intolerance, and hypoxemia for more than 6 weeks after HAdV pneumonia. Lung function tests show obstructive impairment, and high-resolution computed tomography shows a mosaic pattern, bronchiectasis, or atelectasis.

### Statistical Analysis

Statistical comparisons were performed using the two-way ANOVA, chi-square test, or Fisher’s exact test. All statistical analyses were performed using GraphPad Prism 6. *P*-values less than 0.05 were considered statistically significant.

## Results

### Characteristics of the Children With Acute Respiratory Infections

**FIGURE 1:**
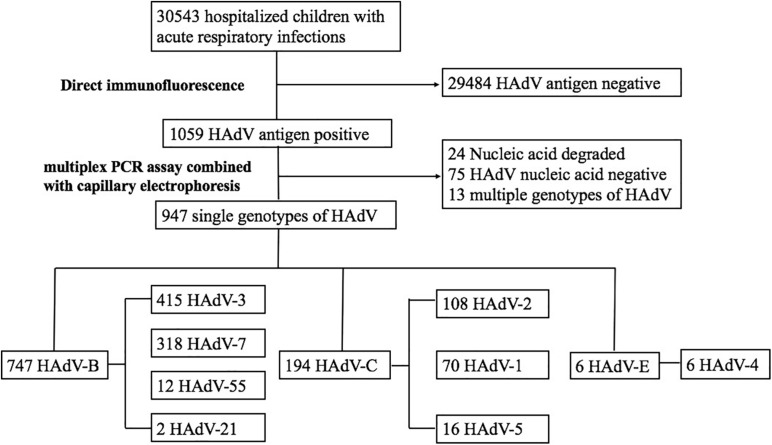
The flow chart of this study.

A total of 30,543 children were hospitalized with ARIs in our hospital. After excluding cases negative for HAdV, non-typed HAdV, and children infected with multiple HAdV types, 947 cases were included in the analysis (Figure 1). Among those children, 600 (63.4%) were male and 347 (36.6%) were female (Table 1). The age ranged from 0.1 to 15.3 years with a median age of 2.9 years, and 488 children (51.5%) were younger than 3 years (Table 1). The main diagnoses were 358 cases (37.8%) of pneumonia, 198 cases (20.9%) of bronchitis, 232 cases (24.5%) of tonsillitis, and 159 cases (16.8%) of URTI.

**TABLE 1:** Demographic and clinical characteristics of children hospitalized with human adenovirus infections (*N* = 947).

Characteristics	*n* (%)
Male gender	600 (66.2)
Age group	
Infant (<1 Year)	116 (12.2)
Toddler (1 Year to <3 Year)	372 (39.3)
Preschool (3 Year to <6 Year)	381 (40.2)
School (6 Year to <18 Year)	78 (8.2)
Pneumonia	358 (37.8)
Bronchitis	198 (20.9)
Tonsillitis	232 (24.5)
Upper respiratory tract infection	159 (16.8)

### Epidemiology and Typing of Hadenoviruses

**FIGURE 2:**
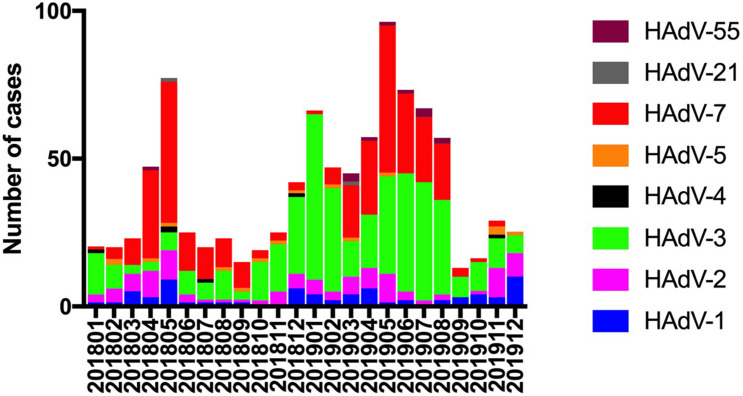
Human adenovirus types detected in pediatric inpatients with acute respiratory infections according to month.

From December 2018 to August 2019, 550 (58.1%) cases of ARIs due to HAdVs were detected. The incidence by month is shown in Figure 2. Phylogenetic analysis revealed that HAdV-3 (415/947), HAdV-7 (318/947), HAdV-2 (108/947), and HAdV-1 (70/947) were the predominant strains. No cases of HAdV-14, HAdV-37, HAdV-40, or HAdV-41 infection were detected. There were 20 cases (2.1%) of coinfection with more than one viral respiratory pathogen. HAdV + human parainfluenza virus-3 (hPIV-3) was the most common type of coinfection (15, 75%), followed coinfections with respiratory syncytial virus (RSV), influenza A virus (Inf A), influenza B virus (Inf B), and hPIV-1 (1, 5% each). One case coinfections with HAdV + hPIV-3 + RSV (1, 5%). The effect of co-infection on the clinical outcomes of HAdV infection cannot be ruled out.

### Human Adenovirus Types According to Age

**FIGURE 3:**
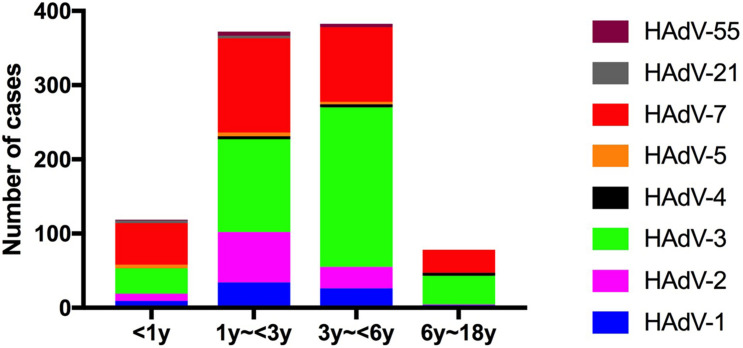
Human adenovirus types detected in pediatric inpatients with acute respiratory infections according to age.

For further comparison, patients were categorized into four age groups: infants (<1 year), toddlers (1–<3 year), preschoolers (3–<6 year) and scholars (6–<18 year), and the HAdV types according to age group were compared. Most affected children were younger than 6 years (Figure 3). The rate of HAdV-7 was higher in infants and toddlers; the incidence of HAdV-2 was higher in toddlers; and the incidence of HAdV-3 was higher in preschool children.

### Human Adenovirus Types According to the Disease Type

**FIGURE 4:**
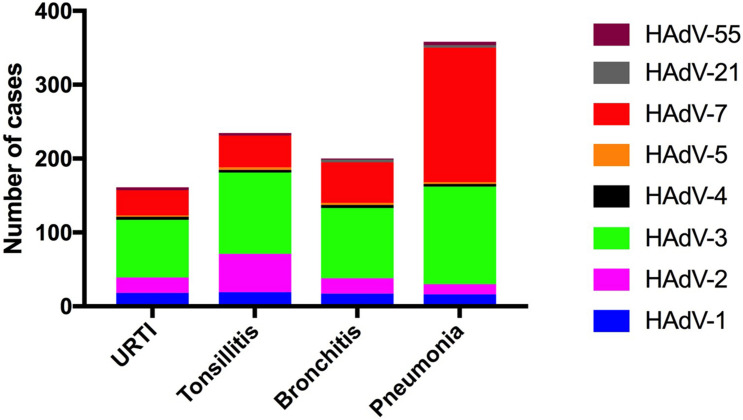
Human adenovirus types detected in pediatric inpatients with acute respiratory infections according to the respiratory disease type.

There were statistically significant differences in the adenovirus types according to the disease type (χ^2^ = 121.9, *p* < 0.0001). For further comparison, the association between HAdV types (HAdV-3, HAdV-7, HAdV-2, and HAdV-1) and disease type was evaluated using Chi-square (112.8, *p* < 0.0001). Among the 358 children with pneumonia, the HAdVs identified were HAdV-7 (*n* = 183, 51.1%), HAdV-3 (*n* = 132, 36.9%), and HAdV-1 (*n* = 16, 2.2%). Among 198 children with bronchitis, the HAdVs identified were HAdV-3 (*n* = 95, 48.0%), HAdV-7 (*n* = 56, 28.3%), and HAdV-2 (*n* = 21, 10.6%). Among the 232 children with tonsillitis, the HAdVs identified were HAdV-3 (*n* = 110, 47.4%), HAdV-2 (*n* = 52, 22.4%), and HAdV-7 (*n* = 43, 18.5%). Among the 159 children with URTIs, the HAdVs identified were HAdV-3 (*n* = 78, 49.7%), HAdV-7 (*n* = 36, 22.6%), and HAdV-2 (*n* = 21, 13.2%) (Figure 4).

### Human Adenovirus Types in Pneumonia

Patients infected with HAdV-3 were older than those with HAdV-7 and other types of HAdV infection. The duration of fever and length of hospital stay were longer among children with HAdV-7. Patients with HAdV-7 were more likely to have severe pneumonia, and had a significantly higher requirement for oxygen. One child died of HAdV-7 pneumonia in hospital. After six-month follow-up, 28 children were diagnosed with bronchiolitis obliterans, including nine children in with HAdV-3, 18 children with HAdV-7, and one child with HAdV-1 (Table 2). Within the follow-up period, no child was diagnosed with bronchiectasis or died post-discharge.

**TABLE 2:** Clinical manifestations and outcomes among children hospitalized with adenovirus pneumonia.

	HAdVs	HAdV-3	HAdV-7	Others	P
Gender (male)	239 (66.76%)	84 (63.64%)	127 (69.40%)	28 (65.12%)	0.547
Age (years)	2.46 ± 1.73	2.81 ± 1.59	2.30 ± 1.89	2.01 ± 1.15	0.000
Underlying disease	28 (7.82%)	11 (8.33%)	16 (8.74%)	1 (2.33%)	0.462
Duration of fever (days)	9.04 ± 4.25	7.98 ± 3.67	10.17 ± 4.23	7.47 ± 4.72	0.000
Wheezing	137 (38.3%)	54 (40.9%)	73 (39.9%)	10 (23.3%)	0.096
Mixed infection	111 (31.0%)	38 (28.8%)	61 (33.3%)	12 (27.9%)	0.619
Mild/Severe CAP	296/62	114/18	142/41	40/3	0.021
Length of stay, days	8.23 ± 5.06	6.86 ± 3.92	9.52 ± 5.44	6.93 ± 5.14	0.000
Admission to PICU	24 (6.7%)	8 (6.1%)	15 (8.2%)	1 (2.3%)	0.432
Patients with oxygen requirement	156 (43.6%)	44 (33.3%)	101 (55.2%)	11 (25.6%)	0.000
Patients with ventilator	3 (0.8%)	0	2 (1.1%)	1 (2.3%)	0.232
Bronchiolitis obliterans	28 (7.8%)	9 (6.8%)	18 (9.8%)	1 (2.3%)	0.247
Death	1 (0.3%)	0	1 (0.5%)	0	1.000

### Phylogenetic Analysis of the Hgenes of HAdV From Hospitalized Children With ARIs

**FIGURE 5:**
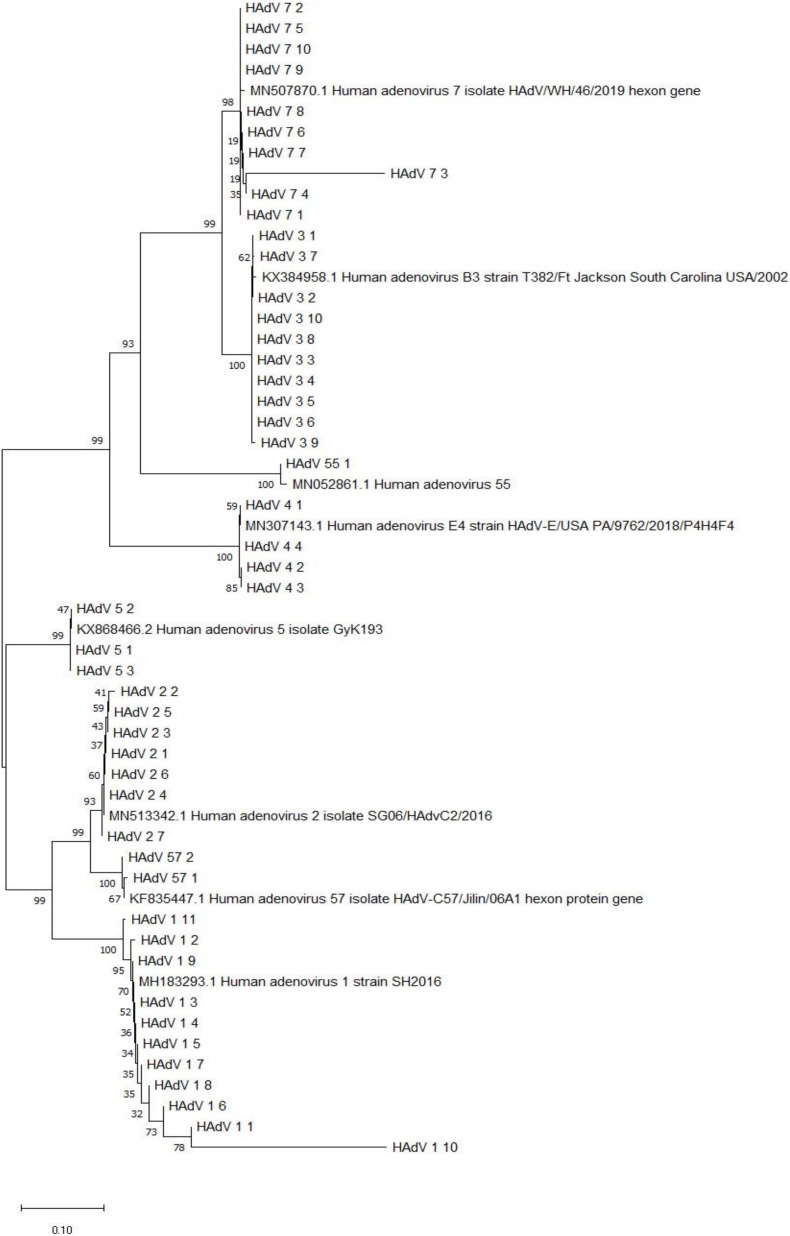
Phylogenetic analysis of the hexon gene from HAdV-positive specimens from 2018–2019. Nucleotide sequences of the archived reference strains were accessible from GenBank.

The sequences of the hexon genes of HAdV specimens from hospitalized children have been submitted to GenBank. Based on the alignment of the nucleotide sequences of the hexon genes with the sequences from relevant strains in China, the phylogenetic tree was built (Figure 5).

## Discussion

Human adenovirus is a major pathogen in hospitalized children with ARIs. In our study, 3.47% of children with ARIs tested positive for HAdV, which is similar to the finding discovery of a study conducted in Hebei (3.71%) during the same period (Zhao et al., 2020). From December 2018 to August 2019, there was an HAdV epidemic in our region. Typing of HAdV is important for understanding the local epidemiology and for vaccine development. From 2012 to 2013, HAdV-3, HAdV-7, and HAdV-55 were the most prevalent HAdV type among hospitalized pediatric patients in Guangzhou (Chen et al., 2016). HAdV-3, -7, -2, and -1 were the predominant types among children in our study. These results are consistent with those of previous studies: HAdV-2, -3, and -7 are the most prevalent types in Beijing and Guangzhou in China (Yao et al., 2019).

Infections with different HAdV-types may exhibit different clinical manifestations (Lin et al., 2015). Most affected children were younger than 6 years. The detection of HAdV-7 was higher in patients younger than 3 years. There were significant differences in HAdV type according to the disease type. The most commonly isolated serotype was HAdV-7 in children with pneumonia, but HAdV-3 was the most common serotype in children with bronchitis, tonsillitis, and URTI. Many studies have shown that HAdV pneumonia is predominantly caused by HAdV-3 and -7 (Zou et al., 2020). HAdV-2 is the most common type isolated in children with tonsillitis. There was a significant difference in types distribution among the different age groups (Chau et al., 2014).

Most adenovirus infections are self-limited, while pneumonia is the most serious disease caused by HAdV infection in children (Shi et al., 2020). The severity of HAdV infection is significantly correlated with serotype (Xie et al., 2018b). Compared with infected HAdV-3, children infected with HAdV-7 had a higher incidence of severe pneumonia, higher requirement for oxygen, and a longer length of hospital stay. Other studies have also found that HAdV-7 was more likely to produce severe pneumonia (Wang et al., 2016; Lin et al., 2017; Xie et al., 2018a). In another study, HAdV-7 infection in pediatric pneumonia tended to have more severe clinical consequences (Liu et al., 2015). A possible explanation for this is HAdV-7 replicates more efficiently, and promotes cytokine dysregulation, causing more severe airway inflammation (Fu et al., 2019; Chen et al., 2020). Yu et al. found that HAdV-7 caused higher pneumonia rates, mechanical ventilation, and a higher fatality rate (28.6%) than other types, particularly HAdV-3 and HAdV-2 (Yu et al., 2016). However, children infected with HAdV-7 do not necessarily have worse clinical outcomes (Zhao et al., 2020). Compared with children with single *Mycoplasma pneumoniae* pneumonia, children with *M. pneumoniae* pneumonia with HAdV coinfection tend to have relatively more severe disease (Gao et al., 2020). Patients with underlying neurologic diseases and prematurity are risk for severe HAdV infections (Cheng et al., 2017).

A high incidence of sequelae and mortality in previously healthy children after HAdV infection was observed in Argentina (Murtagh et al., 2009). In our study, one child died of HAdV-7 infection. Children with HAdV-3 and -7 infection were more likely to develop bronchiolitis obliterans during the 6-month follow-up period. Children admitted with severe HAdV pneumonia, and 22% developed respiratory complications (Li et al., 2018). A family history of asthma, needs invasive or non-invasive ventilation, and infected HAdV-7 are independent predictors of respiratory complications (Li et al., 2018). In another study, they found that a longer duration of fever, dyspnea, or require invasive mechanical ventilation in the acute phase, are rish factors of developing bronchiolitis obliterans in children with severe adenovirus pneumonia (Li et al., 2014; Zhong et al., 2020).

HAdV-5, -55, -4, and -21 were comparatively rare in children with ARIs, and HAdV-14, -37, -40, and -41 were not detected among the children in this study. HAdV-55 as an important pneumonia pathogen in adult in China (Cao et al., 2014), and further surveillance and monitoring of this agent in children with community-acquired pneumonia is warranted. A retrospective stduy conducted in Beijing, they found that HAdV-55 circulated during the spring, and appeared to be as severe as HAdV-7 infections pediatric patients with ARIs (Xu et al., 2018).

This study has some limitations. The children were all hospitalized with ARIs, and the study did not include outpatients. Virus detection using the direct immunofluorescence antibody method is limited due to the limited sensitivity of the procedure; therefore, the observed positivity rates may have been underestimated. Targeted hypervariable regions 1–6 of the hexon gene, HAdV, were molecularly typed by nested PCR amplification and sequencing. Recombination appears to play another novel and major role in the molecular evolution of HAdVs and the genesis of human pathogens. However, the recombinant types were not identified by the adenovirus typing test kit. For the genome recombination analysis, whole genome sequencing and *in silico* restriction endonuclease analysis are recommended to identify genome types (Zhao et al., 2014). In the future research, we will adopt whole genome sequencing and *in silico* restriction endonuclease investigate the molecular evolution of HAdV species.

In conclusion, from December 2018 to August 2019, there was an HAdV epidemic associated with HAdV-3 and -7 in Wenzhou. HAdV-3, -7, -2, and -1 were the predominant types among hospitalized children with ARIs. There were significant differences in adenovirus typing among children with pneumonia, bronchitis, tonsillitis, and URTI. HAdV-7 causes more severe pneumonia in children than HAdV-3.

## Data Availability Statement

The original contributions presented in the study are included in the article/supplementary material, further inquiries can be directed to the corresponding author/s.

## Ethics Statement

The studies involving human participants were reviewed and approved by the Ethics Committee of the Second Affiliated Hospital and Yuying Children’s Hospital, Wenzhou Medical University. Written informed consent from the participants’ legal guardian/next of kin was not required to participate in this study in accordance with the national legislation and the institutional requirements.

## Author Contributions

HZ and XZ contributed to the study design and interpreted the results. YZ, FL, and HL collected the clinical samples. SW and FL collected the clinical data. XZ, LL, and H-HZ performed the experiments. ZL and YZ clinical followed-up. SW and ZL wrote the manuscript. CL and HZ provided critical suggestions on the results and contributed to revision of the manuscript. All authors contributed to the article and approved the submitted version.

## Conflict of Interest

ZX was employed by the company Ningbo Health Gene Technologies Ltd. The remaining authors declare that the research was conducted in the absence of any commercial or financial relationships that could be construed as a potential conflict of interest.
